# Deletion of *MHY1* abolishes hyphae formation in *Yarrowia lipolytica* without negative effects on stress tolerance

**DOI:** 10.1371/journal.pone.0231161

**Published:** 2020-04-03

**Authors:** Oliver Konzock, Joakim Norbeck

**Affiliations:** Division of Systems and Synthetic Biology, Department of Biology and Biological Engineering, Chalmers University of Technology, Göteborg, Sweden; CNR, ITALY

## Abstract

There is a need for development of sustainable production processes for production of fats/oils and lipid derived chemicals. The dimorphic oleaginous yeast *Yarrowia lipolytica* is a promising organism for conversion of biomass hydrolysate to lipids, but in many such processes hyphae formation will be problematic. We have therefore constructed and compared the performance of strains carrying deletions in several published gene targets suggested to abolish hyphae formation (*MHY1*, *HOY1* and *CLA4*). The *MHY1*-deletion was the only of the tested strains which did not exhibit hyphae formation under any of the conditions tested. The *MHY1*-deletion also had a weak positive effect on lipid accumulation without affecting the total fatty acid composition, irrespective of the nitrogen source used. *MHY1* has been suggested to constitute a functional homolog of the stress responsive transcription factors *MSN2/4* in *Saccharomyces cerevisiae*, the deletion of which are highly stress sensitive. However, the deletion of *MHY1* displayed only minor difference on survival of a range of acute or long term stress and starvation conditions. We conclude that the deletion of *MHY1* in *Y*.*lipolytica* is a reliable way of abolishing hyphae formation with few detectable negative side effects regarding growth, stress tolerance and lipid accumulation and composition.

## Introduction

The consumption of vegetable oils is increasing, both for food/feed and for technical purposes. However, this is increasingly leading to a clear conflict with the change in land use for the culturing of oil-crops, e.g. the palm oil plantations, which supply roughly 25% of all vegetable oil, have been associated with environmental issues related to degradation of rain-forest [1]. In addition to the environmental issues, there is also a shortage of suitable areas for cultivating more specialized oil crops (e.g. cocoa) in the face of increasing demand. Thus, there is a strong need for development of sustainable production processes for production of fats/oils and lipid derived chemicals.

A promising strategy is the use of microbial processes to produce fat from hydrolysates of biomass. Several yeast species can accumulate more than 20% of their dry weight as storage lipids, and are therefore characterized as oleaginous yeasts [2]. The most well studied of these oleaginous yeasts is *Yarrowia lipolytica* [3], which has become a popular model production organism for oil/fats and fatty acids, but also for organic acids and proteins/enzymes. *Y*. *lipolytica* can utilize multiple carbon sources and notably also tolerate a wide range of pH and salinity levels (reviewed in [3–5]). An added benefit is that *Y*. *lipolytica* is also generally regarded as safe (GRAS) [6].

*Y*. *lipolytica* is characterized as a yeast, but like many other yeast species it is dimorphic, i.e. it can switch to filamentous growth, forming hyphae when exposed to stress of various kinds, e.g. temperature, pH, mechanical stress, osmotic pressure, carbon and nitrogen source [7]. For large-scale industrial applications this behavior is highly unfavorable as the cell morphology has different effects on the culture rheology, and changes in cell properties can risk the success of the cultivation [8]. Additionally, there are indications that filamentation has negative impact on lipid production in *Y*. *lipolytica* [9]. It would therefore be advantageous to abolish the dimorphic growth, but this should not have negative side effects on e.g. lipid formation or stress tolerance.

Several studies have suggested gene targets for abolishing hyphae formation [10–13]. However, these papers did not address the stress tolerance of the mutated strains. In this paper we therefore constructed several strains with single gene deletions to evaluate hyphae formation, lipid accumulation, fatty acid composition, and tolerance to both long term and acute stress. Of the three proposed gene deletions, only the deletion of MHY1 consistently abolished hyphae formation under all tested conditions. The MHY1-deletion also did not affect growth, lipid production/composition, nor was it more stress sensitive than the wild type strain. In summary these findings indicate that the MHY1-deletion is a suitable modification to achieve non-hyphae-forming strains of *Y*. *lipolytica*.

## Materials and methods

### Strains and strain construction

All strains in this study (see Table 1) are derived from the *Yarrowia lipolytica* strain ST6512 [14]. ST6512 is in turn derived from the W29 background strain (Y-63746 from the ARS Culture Collection, Peoria, USA; a.k.a. ATCC20460/CBS7504) and has been engineered to harbour a KU70::Cas9-DsdA as previously described [15].

**Table 1:** *Yarrowia lipolytica* strains with genotype.

Strain	Genotype
ST6512	W29 MATa *KU70*::Cas9::DsdA
OKYL029	ST6512 *MHY1*-deletion
OKYL030	ST6512 *HOY1*-deletion
OKYL031	ST6512 *CLA1*-deletion

For the construction of gRNA plasmids for the CRISPR-Cas9 driven gene deletion the EasycloneYALI toolbox was used [15]. Primer and gRNA sequences can be found in the supplementary data. The repair fragments were constructed from equal amounts of two single stranded oligonucleotides (around 100 bp; 100 pmol/μl; sequence details provided in the supplementary data) which were incubated for 5 min at 95°C and allowed to cool down to room temperature.

Transformation of *Yarrowia lipolytica* was performed using a lithium-acetate based heat shock method previously described by [16]. Briefly, the strain was plated on YPD agar plates and grown at 30°C for 18h. The cells were washed off the plate and washed twice with water, after which a pellet of 3 OD units in 1 ml, corresponding to roughly 1.6 x 10^7^ cells, was used for transformation. 3 μL of repair fragment (50 pmol/μL) and 500 ng of sgRNA plasmid were added to the washed cell pellet and the cells were carefully resuspended in transformation mix (sterile PEG (43,8% v/v), lithium acetate (0,1 M), boiled single stranded DNA from salmon testes (0,25 mg/mL) and sterile dithiothreitiol (DTT) (100 mM)) and incubated at 39° for 1h. The cells were spun down and resuspended in YPD media which was incubated at 30°C at 200 rpm for 2h. Afterwards, cells were spun down and were resuspended in water before plating on agar plates containing 250 mg/L Nourseothricin (NAT). After 3–4 days of incubation at 30°C colonies could be screened via colony PCR. Colony PCR of transformed *Y*. *lipolytica* was performed by resuspending a colony in 30 μL of 20 mM NaOH, incubating the mixture at 98°C for 15 min, and vortexing the cells together with a small amount (~ 20μl) of 0.5 mm glass beads for 15 seconds. The cell debris was spun down for 1 min at 15000 rpm and 1μL of supernatant was used as template for PCR.

### Media and growth conditions

Lipid production media (LP) consisted of 1.5 g/L yeast extract, 0.85 g/L, casamino acids, 1.7 g/L Yeast Nitrogen Base without amino acids and ammonium sulfate, 5.1 g/L potassium hydrogen phthalate buffer adjusted to pH 5.5, 100 g/L glucose, and either 0.5 g/L urea (henceforth termed LPU-medium) or 0.89 g/L ammonium chloride (henceforth termed LPN-medium) [17].

YPD plates contained 20 g/L peptone from meat, 10 g/L yeast extract, 20 g/L glucose, and 20 g/L agar.

LB plates contained 10 g/L peptone from casein, 10 g/L NaCl, 5 g/L yeast extract, 16 g/L agar, and were set to pH 7.0 with 5M NaOH.

Delft medium consisted of 5 g/L (NH4)_2_SO_4_, 14.4 g/L KH_2_PO_4_, 0.5 g/L MgSO_4_·7H_2_O; adjusted to pH6 with KOH; after autoclavation 2 mL tracer metal solution (FeSO4·7 H_2_O 3.0 g/ L, ZnSO_4_•7 H_2_O 4.5 g/ L, CaCl_2_•2 H_2_O 4.5 g/ L, MnCl_2_·4H_2_O 1 g/ L, CoCl_2_·6 H_2_O 300 mg/ L, CuSO_4_·5H2O 300 mg/ L, Na2MoO4·2 H_2_O 400 mg/ L, H_3_BO_3_ 1 g/ L, KI 100 mg/ L, Na_2_EDTA•2 H_2_O 19 g/ L), 1 mL vitamins (d-Biotin 50 mg /L, D-Pantothenic acid hemicalcium salt 1.0 g/ L, Thiamin-HCl 1.0 g/ L, Pyridoxin-HCl 1.0 g/ L, Nicotinic acid 1.0 g/ L, 4-aminobenzoic acid 0.2 g/ L, myo-Inositol 25 g/ L), and 20 g/l glucose were added.

YNB media consisted of 1.7 g/L yeast nitrogen base (without amino acids), 1.5 g/L yeast extract, and 50 g/L glucose [18].

All *Y*. *lipolytica* strains were cultivated in suspension at 30°C in 10 mL media in 100 mL Erlenmeyer flasks at 200 rpm shaking unless differently stated.

For the visual confirmation of hyphenation, the strains were grown in different liquid media or agar plates. At different time points an aliquot of cells were diluted and checked for hyphenation under a bright field microscope.

*Escherichia coli* DH5alfa was cultivated in LB broth or on agar plates supplemented with 100μg/mL ampicillin at 37°C. Plasmid construction was conducted following the USER cloning protocol as previously described [15].

### Lipid extraction and quantification

*Y*. *lipolytica* strains were cultivated in LPU or LPN media for 96 hrs before fatty acid methyl ester (FAME) extraction to measure cellular lipid content. The protocol used was previously described by [19]. In short, 100 μl of cell culture was spun down, the supernatant was discarded, and the cells were washed with 1mL water. Then 40 μg of C17:0 internal standard was added to the cell pellet. 500 μL of methanol solution containing 1M NaOH was added and the samples were vortexed at 1200 rpm at room temperature for 1h. The solution was neutralized by carefully adding 80 μL of 50% sulfuric acid. The FAME’s were extracted by adding 500 μL hexane. Phases were separated by centrifugation for 1 min at 10.000 xg. 200 μL of the upper hexane phase was mixed with 800 μL hexane and 1 μL of this sample was analyzed on GC-MS (Thermo Scientific Trace 1310 coupled to a Thermo Scientific ISQ LT with a ZBFAME column (Phenomenex, length: 20 m; Inner Diameter: 0.18 mm; Film Thickness: 0.15 um)).

To allow a calculation of lipid content per cell dry weight (g fatty acid / g dry biomas), the dry weight of each culture was calculated as follows: 1 ml of culture was filtered, followed by a wash of the filter with 10 ml of milliQ water prior to drying and weighing the filters. For the calculation of lipid content, C16:0, C16:1, C18:0, C18:1 and C18:2 fatty acids were considered. For the fatty acid composition, the contribution of each fatty acid species to the total fatty acid content was calculated.

### Stress tolerance test

The general setup for testing of stress tolerance of the strains was based on the procedure of Martinez-Pastor *et al* [20] and adapted for *Y*. *lipolytica*.

The tolerance of strains towards high temperatures and oxidative stress was tested by inoculating cultures to OD 0.1 and letting them grow for 6 h to reach mid exponential growth phase. For determination of stress from elevated temperature, aliquots of the cultures were either cultivated at 39°C for 1 h, or at 50°C for up to 8 minutes, with samples taken out at indicated intervals. For determination of oxidative stress tolerance, hydrogen peroxide was added to a final concentration of 50 mM and incubated for 1 h with 200 rpm shaking. After treatment, cells were serially diluted in 1/10 steps, and 5 μL drops from each dilution were placed on YPD plates. Every condition was tested with three independent cultures, and samples from every culture were plated in technical duplicate. After 1 day of incubation at 30°C colony forming units (CFU) were counted, and the viability was calculated by comparing the CFU of treated culture to an untreated control.

For testing nitrogen starvation tolerance, strains were cultivated in LPU or LPN medium for up to 10 days. Drop tests were performed after 3, 5, 7 and 10 days and the colony count of day 3 was used for normalization.

For testing glucose starvation tolerance cells were first grown in LPU media with 100 g/l of glucose. Cells from a stationary, and exponential culture were then used to inoculate 15 mL of LPU medium without any glucose to a starting OD_600_ of 0.3. Drop tests were performed right after inoculation, and daily for up to 7 days.

All chemicals stated in the methods were obtained from Sigma-Aldrich if not stated differently.

For the calculation of significance a two-tailed two sample unequal variance t-test was performed.

## Results and discussion

### Comparison of mutations reported to abolish hyphae formation

Many different conditions promote hyphal growth of *Y*. *lipolytica* [7], which can cause problems with fermentations and cell handling. The ability to abolish hyphae formation in *Y*. *lipolytica*, with minimal alterations to the cell physiology, would therefore be highly desirable. Several genes have previously been identified which upon deletion or over-expression abolish or reduce hyphae formation [10–13]. However, in most of these studies, the possibility of an altered stress sensitivity was not addressed. We therefore decided to delete three of the most promising published candidate genes (*HOY1(YALI1_A19214g + YALI1_A19220)*, *CLA4 (YALI1_C31453)* and *MHY1/YlMSN2(YALI1_B28150g)*) in *Y*. *lipolytica* strain ST6512 (henceforth termed WT or wild type). Briefly, plasmids carrying sgRNA expression cassettes and the NAT-marker, and repair fragments were transformed into *Y*. *lipolytica* WT strain [15]. Single colonies were re-streaked and tested for gene deletion by colony PCR.

The wild type strain was subsequently cultivated on several different conditions for up to 12 days, during which time visual microscopy inspection was performed at several timepoints. In liquid YPD-, YNB- or Delft-medium with glucose or glycerol as a carbon source and ammonium as nitrogen source, the wild-type strain displayed no hyphae formation. It has been previously reported that N-acetylglucoseamine (GlcNAc) induces filamentation of *Y*. *lipolytica* [21]. In the present study, the wild type strain showed only a low level of hyphae formation in Delft medium with glucose and GlcNAc, a phenotype which was abolished in all tested mutant strains (S1 File). We also tested whether growth on GlcNAc as a sole carbon source would lead to hyphae formation. However, no hyphae formation was observed under these conditions, in line with a previous study [22]. Since our wild type strain did not show a high filamentation rate in the tested liquid media, we also decided to investigate if hyphae formation would be observed on solid media. In this experiment we spread cells on LB-plates and YPD-plates, which will differ mainly in the available carbon source, i.e. glucose on YPD-plates and amino-acids on the LB-plates. LB-plates also contain 5 percent sodium chloride which will contribute to a mild osmotic stress. In this case, hyphae formation was clearly observed already after 1 day for both media.

**Fig 1:**
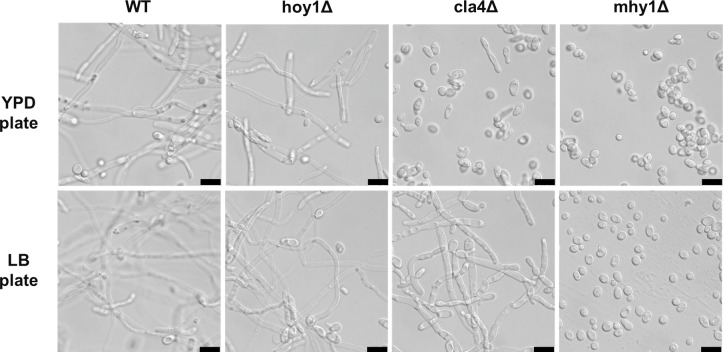
Cell morphology of WT strain and putative hyphae negative strains on solid media. The indicated strains were plated either on YPD or LB plates and cultivated for 4 days at 30°C. Black scale bar equals 10 μm.

The first gene we tested was *HOY1*, which encodes a homeobox-protein transcription factor, which deletion was reported to abolish hyphae formation [13], while its overexpression was reported to increase hyphae formation [23]. We found that the *HOY1*-deletion strain in our background (OKYL030) did not show any reduced hyphae formation (Fig 1). This was highly surprising since the previous identification of *HOY1* as a gene involved in hyphae formation was also performed in the W29 background of *Y*. *lipolytica* [13], which is the same background as that of our wild type strain. Therefore, to make sure that no strain mixing has occurred by accident, we have verified the lack of effect on hyphae formation with the frozen strain, and also by additional PCR-steps with different primer sets, which have all shown that the *HOY1* open reading frame is not present in our *HOY1*-deletion cells. There are two reported sequences of the W29 derived *HOY1* ORF (which differ in the presence of two inserted bases; see Supplement data) one of which will lead to a truncated version of the Hoy1 protein (reported as two separate open reading frames). Our strain background has the *HOY1*-sequence lacking these insertions, thus encoding a full-length Hoy1 protein, and therefore not providing any clue to the mechanism underlying the absence of the expected *HOY1*-deletion phenotype. We conclude that the *HOY1*-deletion is not optimal for abolishing hyphae formation in *Y*. *lipolytica*, due to its apparent sensitivity to strain background.

Next, we tested the deletion of *CLA4*, which encodes a protein kinase, and was reported to abolish the ability to hyphenate or to invade agar [12]. The *CLA4*-deletion strain (OKYL031) on YPD-plates did not show clear formation of filaments, but a few elongated cells were observed (Fig 1, upper panel), perhaps related to a defect in its reported role in cell polarity maintenance [12]. However, on LB-plates, a clear hyphal growth was observed (Fig 1, lower panel). Therefore, we conclude that deletion of *CLA4* does not seem to be a reliable way of abolishing hyphae formation.

Finally, we tested the deletion of the *MHY1* gene, which has a C2H2-zinc finger, DNA-binding domain on the C-terminal. Deletion of *MHY1* has been shown to abolish hyphae formation [11, 24–26], while overexpression increased hyphae formation [11]. In our hands, the *MHY1*-deletion (OKYL029) was the only strain which abolished hyphae formation under all tested conditions (Fig 1). Thereby suggesting it as the most suitable candidate gene to accomplish the goal of a *Y*. *lipolytica* strain always growing in the yeast form.

### Effect of mhy1-deletion on lipid accumulation and composition

**Fig 2:**
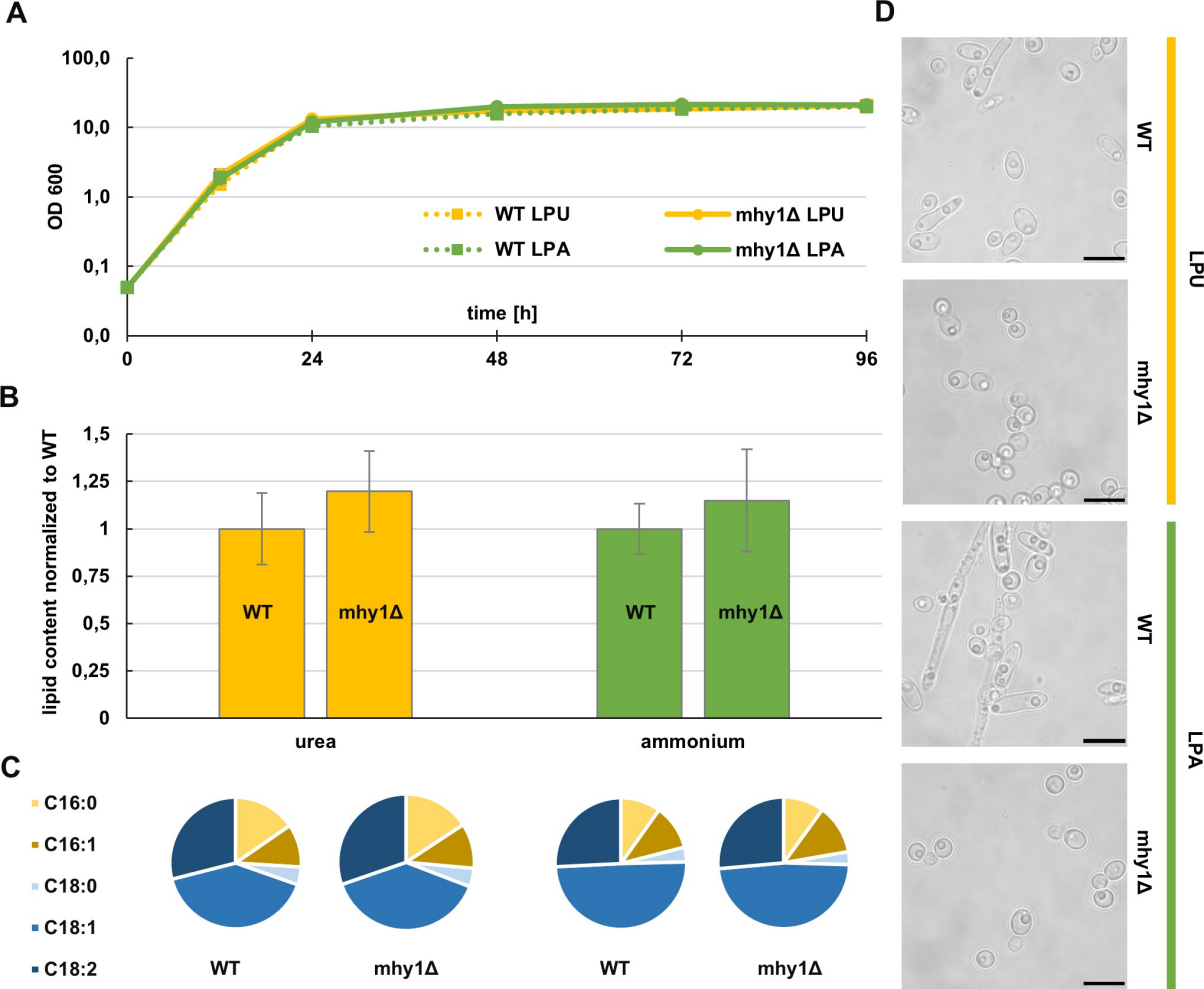
Influence of *MHY1*-deletion on lipid content and composition. Strains were grown in LPU (urea) or LPA (ammonium) medium for 4 days before performing FAME extraction. (A): growth curve of the two strains (B): cell lipid content of strains in different media normalized to the WT. (C): total fatty acid composition of the strains in different media. For the calculation C16:0, C16:1, C18:0, C18:1 and C18:2 fatty acids were considered, displayed is the contribution of each fatty acid species to the sum of all. (D): Microscope images of the cells at the moment of harvest, the size of the black bar equals 10 μm. Bars and error bars represent mean of biological triplicates and standard deviation respectively.

*Y*. *lipolytica* is both a model organism for oleaginous yeasts and a promising host for industrial scale lipid production, and much research has been performed to increase and manipulate its lipid content and fatty acid composition. It would be a strong disadvantage if the *MHY1*-deletion would have a negative impact on the lipid accumulation and composition. We therefore also investigated in the effect of the *MHY1*-deletion on the lipid content and the fatty acid composition. Since the nitrogen source used can affect the lipid production [27], we decided to test the *MHY1*-deletion with two different nitrogen sources, urea and ammonium, both of which are likely to be used in a production process. Both strains showed a similar growth profile (Fig 2A). We observed that the *MHY1*-deletion strain accumulated somewhat more lipid than the wild type in both nitrogen sources tested (Fig 2B), in line with previous findings [25, 26], further strengthening the beneficial traits of the *MHY1*-deletion. We also studied the fatty acid composition of the two strains, which did not show any significant changes (Fig 2C, detailed in supplement Table 1).

As can be seen in Fig 2C, we observed a clearly different fatty acid composition depending on the nitrogen source, which was not dependent on the absence or presence of *MHY1*. In urea-based medium there was more C16:0 and C18:2, and less C18:1. We also observed that, for both the WT and *MHY1*-deletion strains, the specific lipid content (g/gDW) was higher in the LPA-medium than in LPU-medium.

In both LPA- and LPU-medium, the *mhy1*-deletion was found to abolish the weak filamentation observed in the wild type (Fig 2D).

### Acute stress tolerance of MHY1-deletion strains

It has been suggested, experimentally verified, and predicted from the C2H2-domain sequence homology that Mhy1 would bind to ^5’^CCCCT ^3’^ or ^5’^ AGGGG ^3’^, suggesting it to be a functional homolog of the C2H2-trancription factors *MSN2*, *MSN4* and *COM2* in *S*. *cerevisiae* [11, 24]. All the *S*. *cerevisiae* homologs of *MHY1 (MSN2*, *MSN4* and *COM2*) are reported downstream targets for regulation by the RAS-cAMP, protein kinase A-pathway, and especially for Msn2/4 regulation it has been shown to be strongly dependent on RAS-pathway. It has also been suggested that Mhy1 functions downstream of Ras2, controlling the dimorphic switch via a novel Ras signaling pathway, where a high PKA activity would antagonize the hyphae formation [28, 29].

*MSN2* and *MSN4* in *S*. *cerevisiae*, are the main effectors of the general transcriptional stress response [30], and consequently the *msn2/4*-deletion mutant showed an increased sensitivity towards heat, osmotic and oxidative stress [20]. In view of the similarity in target promotor motif and protein kinase A regulation we hypothesized that a deletion of *MHY1* would lead to stress sensitivity, which would be a strong drawback for the use of non-hyphae-forming, *mhy1*-deletion mutants, e.g. in an industrial setting. Consequently, we decided to investigate if the deletion of *MHY1* in *Y*. *lipolytica* would have a similar effect on the stress sensitivity as an *msn2/4*-deletion in *S*. *cerevisiae*. To this end, *Y*. *lipolytica* wild type and *MHY1*-deletion strains were tested for survival under acute stress conditions (temperature and oxidative stress) and long-term starvation (nitrogen or glucose limitation).

**Fig 3:**
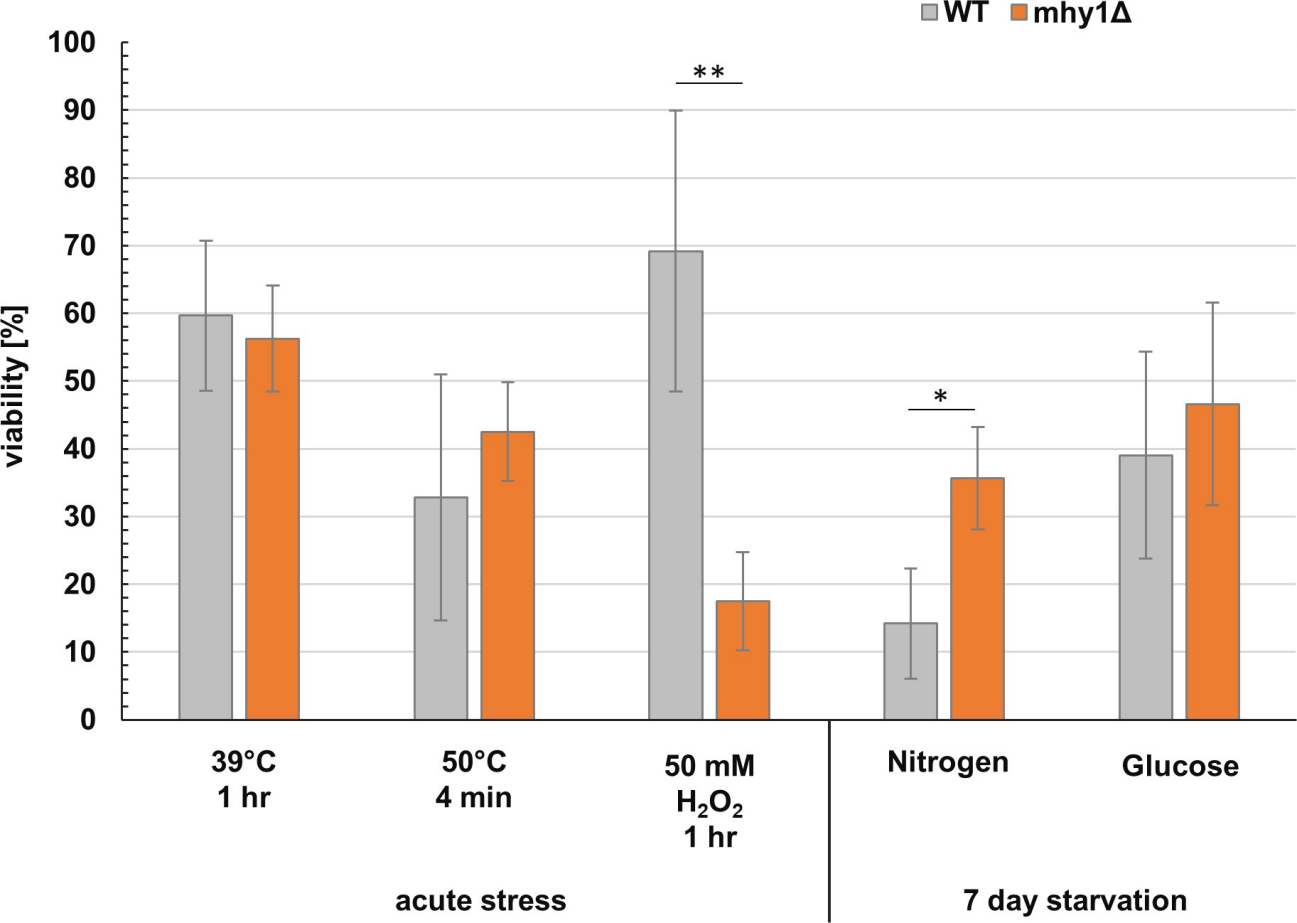
Stress tolerance in temperature, oxidative stress and starvation. Acute stress: cell survival after 1h at 39°C, 4 min at 50°C or 1h with 50 mM H_2_O_2_, viability as % of CFU before treatment. Starvation: cell survival after 7 days of nitrogen or glucose starvation, viability as % of CFU after 3 days. Bars represent the mean of biological triplicates plated in technical duplicates; error bars represent standard deviation. WT-cells in grey, *MHY1*-deletion cells in orange. For the statistical analysis a * indicates a p-value < 0.05 and ** p-value < 0.01.

We first tested whether exposure to elevated temperatures would lead to a more rapid loss of viability in an mhy1 deletion. We chose two different temperatures for testing: A modest temperature increase up to 39°C for 1 hour (this is the common conditions for heat shock transformation of *Y*. *lipolytica*), and a severe temperature shift to 50°C, which killed almost all cells of both strains within 10 min (S1 File). The *MHY1*-deletion strain showed a similar, or even slightly better, survival rate as the wild type strain under both moderate and severe temperature stress (Fig 3).

We next tested whether an *MHY1*-deletion would cause an increased oxidative stress sensitivity. A range of H_2_0_2_-concentrations was tested, and as previously observed [31, 32], *Y*. *lipolytica* was found to be highly tolerant to this type of stress, with levels of 50 mM H_2_O_2_ for 1 hour needed to achieve a reduced viability for the wild type of 69%, compared to non-stressed cells (Fig 3). Under the same conditions, it was observed that the *MHY1*-deletion strain showed a significantly lower survival rate (p<0.01) compared to the wild type, with 18% of the cells surviving compared to non-stressed cells (Fig 3). However, this is only observed at concentrations of H_2_O_2_ which it is highly unlikely that *Y*. *lipolytica* cells would encounter in an industrial setting. A previous paper states that a *MHY1*-deletion strain was not stress sensitive, based on a lack of growth effect from a very low level of H_2_O_2_-stress (0.5 mM) or 1 M NaCl [33]. At our severe H_2_O_2_-stress levels, the difference in sensitivity of the wt vs the *MHY1*-deletion strain was much less prominent than that observed when comparing a wild type *S*. *cerevisiae* strain to a strain deleted for *MSN2/4* [20].

From the above experiments, the fact that an *MHY1*-deletion does not share phenotypic traits with a *MSN2/4* deletion in *S*. *cerevisiae* leads us to the conclusion that Mhy1 is not a functional homolog of Msn2/4, even if it seems to bind the same promotor motif. It has been suggested to rename *MHY1* to *YlMSN2* [24]. However, since there is no significant homology to the ScMSN2/4 and other C2H2 zinc finger proteins outside of the C2H2 domain, and since there is little indication for a role of Mhy1 in stress tolerance, we propose to keep the name *MHY1*.

### Starvation stress tolerance of MHY1-deletion strains

One of the main process applications of *Y*. *lipolytica* is lipid production, which usually involves prolonged starvation for nitrogen. It would be a strong disadvantage if the *MHY1*-deletion would confer starvation sensitivity. We therefore tested the long-term starvation survival of the *MHY1*-deletion towards nitrogen and glucose limitation. The colony forming unit count (CFU) of cultures with limiting carbon or nitrogen was compared for the wild type and *MHY1*-deletion strains, over a period of 7 days, with the survival percentage expressed as values normalized to the CFU after 3 days (72 hr) starvation. The conclusion was that the *MHY1*-deletion survived equally well, or even better than the wild type strain, both under nitrogen and carbon starvation conditions (Fig 3). Thus, providing further evidence for the beneficial role of the *MHY1*-deletion in biotechnological processes.

## Conclusion

We have shown that a deletion of *MHY1* is the most reliable way of abolishing the formation of hyphae in *Y*. *lipolytica* under several conditions. We could also confirm that the deletion of *MHY1* mediates a small increase in the lipid content of the cells, without any observable a change in fatty acid composition.

The *MHY1*-deletion conferred a minor increase in the sensitivity towards severe oxidative stress but did not increase the heat stress sensitivity or long-term survival under glucose or nitrogen limiting conditions.

In conclusion, our findings together with previous reports suggest that the *MHY1*-deletion is a modification to consider in most *Y*. *lipolytica* based applications in which filamentation could pose a problem.

## Supporting information

S1 Fig Showing the GlcNAc effect on WT and mutant strains. (DOCX)

S2 Fig Showing results of a pilot experiment of heat tolerance of ST6512 at 50°C. (DOCX)

S1 Table With the lipid composition data for Fig 2. (DOCX)

S1 File DNA-sequences of primers, repair fragments and target genes. (DOCX)
