# Supplementary material for: Deletion of MHY1 abolishes hyphae formation in Yarrowia lipolytica without negative effects on stress tolerance
Source: PLoS One. 2020 Apr 3;15(4):e0231161. doi: 10.1371/journal.pone.0231161 (PMC7122783; doi:10.1371/journal.pone.0231161)
Supplement: S1 Fig — (DOCX) [file pone.0231161.s001.docx]

**Supplement figure 1**

**Cell morphology of WT strain and putative hyphae negative strains on GlcNac-media.** The indicated strains were cultivated for 4 days at 30°C in Delft medium with glucose and N-acetyl-glucosamine (GlcNAc). Black scale bar equals 10 µm.
