# Supplementary material for: Deletion of MHY1 abolishes hyphae formation in Yarrowia lipolytica without negative effects on stress tolerance
Source: PLoS One. 2020 Apr 3;15(4):e0231161. doi: 10.1371/journal.pone.0231161 (PMC7122783; doi:10.1371/journal.pone.0231161)
Supplement: S2 Fig — (DOCX) [file pone.0231161.s002.docx]

**Supplement figure 2**

**Cell survival of ST6512 incubated in 50°C.** Cells were grown in LPU media for 14h (exponential phase) and then incubated at 50°C for 1 to 10 minutes before plating on YPD plates in different dilutions. CFU were counted after 1 day of incubation at 30°C and viability was calculated by comparing CFU of treated to an untreated control.
