# Supplementary material for: Deletion of MHY1 abolishes hyphae formation in Yarrowia lipolytica without negative effects on stress tolerance
Source: PLoS One. 2020 Apr 3;15(4):e0231161. doi: 10.1371/journal.pone.0231161 (PMC7122783; doi:10.1371/journal.pone.0231161)
Supplement: S1 Table — (DOCX) [file pone.0231161.s003.docx]

**Supplement table 1**

**Data for figure 2.** Percentage of each fatty acid in the FAME extraction lipids for the indicated media and strains.

| **Medium** | **Strain** | **C16:0** | **C16:1** | **C18:0** | **C18:1** | **C18:2** |
| --- | --- | --- | --- | --- | --- | --- |
| LP-urea | WT  MHY1∆ | 15.4 ± 0.4  15.7 ± 0.4 | 10.6 ± 0.5  10.7 ± 0.5 | 4.4 ± 0.5  4.4 ± 0.4 | 40.7 ± 1.5  38.9 ± 0.8 | 29.0 ± 1.5  30.3 ± 0.1 |
| LP-ammonium | WT  MHY1∆ | 9.9 ± 0.7  10.0 ± 0.3 | 11.1 ± 0.5  12.3 ± 1.4 | 3.6 ± 0.3  3.2 ± 0.1 | 49.6 ± 2.5  48.1 ± 2.1 | 25.8 ± 2.5  26.4 ± 3.7 |
