# Supplementary material for: Deletion of MHY1 abolishes hyphae formation in Yarrowia lipolytica without negative effects on stress tolerance
Source: PLoS One. 2020 Apr 3;15(4):e0231161. doi: 10.1371/journal.pone.0231161 (PMC7122783; doi:10.1371/journal.pone.0231161)
Supplement: S1 File — (DOCX) [file pone.0231161.s004.docx]

**DNA oligos**

For construction of gRNA plasmids

Construction was done via the protocol of EasycloneYALI [1]. Marked in orange are USER cloning overhangs, black are the 20 nt CRISPR recognition sites.

| MHY1_cut-site_1_FW | aggcgacagcatgtaaatgggttttagagct |
| --- | --- |
| MHY1_cut-site_1_RV | ccatttacatgctgtcgccttaaccaacct |
| MHY1_cut-site_2_FW | ctcgagaggcgagtaagtgggttttagagct |
| MHY1_cut-site_2_RV | ccacttactcgcctctcgagtaaccaacct |
| HOY1_cut-site_1_FW | cgtggatgtgaggttggcgggttttagagct |
| HOY1_cut-site_1_RV | ccgccaacctcacatccacgtaaccaacct |
| HOY1_cut-site_2_FW | tcgtgtggcagcgtcgggaggttttagagct |
| HOY1_cut-site_2_RV | ctcccgacgctgccacacgataaccaacct |
| CLA4_cut-site_1_FW | gtcatggagtatatggaggggttttagagct |
| CLA4_cut-site_1_RV | ccctccatatactccatgactaaccaacct |
| CLA4_cut-site_2_FW | gtttgtgccgctggaagagggttttagagct |
| CLA4_cut-site_2_RV | cctcttccagcggcacaaactaaccaacct |

Repair fragments for gene deletion

Gene deletion repair fragments were created by mixing deletion_box1 with deletion_box2 in equal amounts and heating up to 95°C for 5 min.

MHY1_deletion_box1 cgccaaaaccatcccaatcgaaaaacaaaaagtcatagtacattatcgccaaaaggtagagcgccttctagtctccgctccatttttttattgtaaccag

MHY1_deletion_box2 ctggttacaataaaaaaatggagcggagactagaaggcgctctaccttttggcgataatgtactatgactttttgtttttcgattgggatggttttggcg

HOY1_deletion_box1 cacggtcagttgaccaatgtagattagtaagcgtccgggttagacatgtggcgctggagggggtggtggtggtggtgacagaggtggagtgg

HOY1_deletion_box2 ccactccacctctgtcaccaccaccaccaccccctccagcgccacatgtctaacccggacgcttactaatctacattggtcaactgaccgtg

CLA4_deletion_box1 gtccaaattccacccatattcaccgttatacctataccgtacccatgtcagtcgggatgtgcaattaactcatggcagtgcgacgctccacgctatac

CLA4_deletion_box2 gtatagcgtggagcgtcgcactgccatgagttaattgcacatcccgactgacatgggtacggtataggtataacggtgaatatgggtggaatttggac

Primer for screening for successful deletion

| MHY1-del_FW_Screen | tctttcaaccgtcccacacc |
| --- | --- |
| MHY1-del_RV_Screen | caaagtggacaagcgactgc |
| HOY1-del_FW_Screen | aacgcaccaactaacgaccc |
| HOY1-del_RV_Screen | aactccctttacctggacgc |
| CLA4-del_FW_Screen | cacatcccccaactctttgc |
| CLA4-del_RV_Screen | tgcgactatgaggtgatggg |

Expected length of PCR product for either wild type or deletion of the target genes:

| Gene | Expected wild type band [bp] | Expected deletion band [bp] |
| --- | --- | --- |
| MHY1 | 1533 | 675 |
| HOY1 | 2186 | 654 |
| CLA4 | 3532 | 598 |

**Gene sequences**

**MHY1**

YALI1_B28150g (aka YALI0B21582g) atggacctcgaattggaaattcccgtcttgcattccatggactcgcaccaccaggtggtggactcccacagactggcacagcaacagttccagtaccagcagatccacatgctgcagcagacgctgtcacagcagtacccccacaccccatccaccacaccccccatttacatgctgtcgcctgcggactacgagaaggacgccgtttccatctcaccggtaatgctgtggcccccctcggcccactcccaggcctcttaccattacgagatgccctccgttatctcgccatctccttctcccactagatccttctgtaatccgagagagctggaggttcaggacgagctcgagcagcttgaacagcagcccgccgctctctccgtcgaacatctgtttgacattgagaactcatcgatcgagtatgcacacgacgagctgcatgacacctcttcgtgctccgactcgcagtcgagcttttcccctcagcagtcccctgcctccccggcctccacttactcgcctctcgaggacgagtttctcaacttggctggatccgagttgaagagcgagcccagcgcggacgacgagaaggatgatgtggacacggagcttccccagcagcccgagatcatcatccctgtgtcgtgccgaggccgaaagccgtccatcgacgactccaaaaagacttttgtctgcacccactgccagcgtcggttccggcgccaggagcatctcaagcgacatttccgatccctacacactcgagagaagcctttcaactgcgacacgtgcggcaagaagttttctcggtcggacaatctcgcccagcatatgcgtacgcatcctcgggactag

**HOY1**

YALI1_A19214g + YALI1_A19220 (aka YALI0A18469g)

**ATG**GACAAGAAACGATCAAAGAAGTCGACGCTGACACAGCAACAACGCAACAACAAGCGCCAGAGAGCTAACGCACAGCAATTGGACGTGCTCCGCCACGAGTATCGTCTGTGTGCCACTCCCGACGCTGCCACACGACGTCGAATAAGTGCTCTTATCGATATGACTGAGCGTAGCGTTCAGATTTGGTTCCAGAACACGCGTGCCAAGCAGAAGAAGGCTATGCGCGGTCGGGAATCTCGCGAGGACGACTTTGACGACGGTCTTGCTGATGTCAGCGTGGATGACGTCAGCGTGGATGACGTAAGCATGACGGCTGACGTAATCAACAGTCCTGTTGACGTAATCCACCATCGCACCGACGTTAGCAGTTCCGCAGACATGCTCCACCACTTTGGCGCCGACCTCGTCACAACCCCCATCCAAACTCATTTCAACCACTTGCTCGCAACCCCACCAACCTCTTCCTCGTCGTCGCACGTGACCACGGTTGACTCACCCGCCTCGTCCATCACCGACTTCTCCACCCTCGGACACATGACTCCTAACCTGACCCCCACCACACCGCATCAACGT**TGA**CCCTCCCGCCCATCTTCTTCCCCACCCTTTCGCTGACTATTGGAAATTGGCGCCGACTTTCGCCCCAACTTTCTCTTGCTTACTACCCCAGCACAGACACC**ATG**CTGTATCACATGACCTCGGAGAAGACGCAGTTTCGCATGCAGTTTCCCTTTTCGGCGATTGAGGAAATCCACGTGAGCAGAAACCCCAACGATACTTTCGGAGCTCTTAATCTGACGCTCAATTGCTCACCCAGCTTTTCGATCCAGACCCCAAAGGCTCCAGGAAGATGGGTCGGCTGTCACGACTTTTCCGAGGGCAAACAAGCGAGTAACGTGACTACTCACGTGTGCAACGGTCCCGCAACGGTGTTGCAACAGCAGCTTTCGCGGGTGTTTGCCCTGCGCAGCAACCATGTGAACGGTCACGTGTCACGACGACGGCTCGCGGACGTCATTGGGTCTTCTGACATAATCGGATCTGCTGACGTAATGGGGACTGTCGCCGTCGACACCTCCAACCACATGATCTCCAGTCATGTGGGCGACTTTGAGCTGAGCGTGACCAGCCACTGTGAGCCGTCCCACATGATCGAACCGACGCCGACGCCGACGCCAGTGGCCAACCACCACCATCATCGATCTGCTGACTCAGTCGCTCACGTGACAAACCCTAGTGGCCACGTGACCAACCATAACCACCTGTCCGACCCCGCCGCCAACCTCACATCCACGATTCTCGGAAACGTGACGCCAGGACACACGACACCGGTTCTGGACGACGGGTTCGAGCTCGGTCACGTGACTGACATCAACGACCTCGTCAGTGTTGCGCAATTGTTGCCAAACCACGTGACCGAGGCCGAGCTCGCGAGCAACTGTCACATGATTGCCAACAATGCCTTTACTTTGGATCCGTCTACTCCTCTGAGTCCGTTTTCGGGCACGTTGGATTATCTGGAGTATTAC**TAA**

Underlined bases are not present in the ST6512 genome. The two orf’s are one orf.

**CLA4**

YALI1_C31453 (aka YALI0C22770g) atgaaaaatcccgaccatgggcaataccaaactttccatgatgaagacgctacgacacacacagctgtccaaggagcccacattcagctcatgatgtatgcagcttctcctgtcgtccctacattcgagatgctcgcaacactcatttcgaagactgtactaactcagatgtcacaattcagccaagcatttctaaaccctggtcctgccccaccgccacccaagcccttcgccagtagcctcaccgcctcttccagcggcacaaacctttccgggctcatgacccccacagagccccgtaccgcccccaccatcacacgagacgccatcacacgacccgcgtcgtccaacagggtgctcaagtccgggttcgcgtccgtcaaggaggatggatcatcgttccggtcgctcatgtggactaaaaagtacttggtgttgcgggaaaatgcattggactttcaaaagtcggagacgtcgtcagtgcagttctccatcccgctatacacggtctcgaacgtgacacgtgtcgatctcaagccatattgcttcgagattgtgcggtcgtcgggcttcaagtccgtgttcgtcgtctgcaagaatgacacggagctgtacgcctggatcgatgagatctactccaaatgcccactcatgggcgtctctaatcccaccaacttcacacataaggtgcacgttggcttcgaccccatctccggaggcttcactggtctacccgacgcctggagcaagctgctttcgacgtcagccatcaccaaggaagactacgccaagaaccctcaggccgtcatcgaggtgcttgagttctacagcaaggaaaatgttgaggccatgggattcaacgcatcttctttgggcattggcattgagaacaagtttgatgagggcctgacaatggacgccgctcgtgtgcgggccatgcagcagcagcagaagcagcagcaggcccagcgtatggccaacaacctgcaggagtggaccaaacccgtccctagtactggctcttccagctcttcaacagccaccactcccgcacgatataatgccacacgaaagaacgacgacgatcttctcacgtctctttctccgtccacatccgtcccccgacgaccctcggtccccaacgtcacccgggcggcccccccacggcctgagcgacctgatggactgccatcgacctcgacgatccctagggaccgaagcgagctgcgacaggaactgcgtaacgaaatgcgtgccggtgctggaaacagcagtggaagcgtcagcggagccagtggcagcggcactcagacccccgtgcatcctctgctgcgaatcaaccccgacgttgactcgtaccagcaacgacaatcgcccaccagcaacaccggaggcaactaccccacgacgcccacgtcgatgacccagtcgtcgtcgcagggctctctgtcgtccaagacatcgcccaccgcatacagagcctctcccgtggccacagctgctttgcacgatctaccgcccccccagcgacccatgctgcgacacaagaactccagtggttctatcaactctgtgtcgtcgggaaactcatacgtcgctcctctcatggtcaagaaggccatccctgccaaccggcctcctcagactgcccctccggtgcctgcacagagcggtggagacaaattccagccccagcgagctgctcctgtgcctcagcaaactcagcagccccaggcactccctgcatcttcaggtacgtctgctgctcccgctcttggagcggctaaccagttcaagccccagcggcctgcaccatcgccccagaagacgacccccaagacgtctcccaagaagccctctgctgctggtgtcggccagggtggctacccaagaatgtctcccactaagacgtctcctaccaagacgtctcctggtggagctagtgctgccgctgctgccgccgccgccgctgctacaactgctgctctcgaaggtcccaagaagaagccccagcatgacgagcgtcgaatctcgaccatgaccgacgcacagatcaccgagaagctcaagtcggtggtcaacccccaggaccctactcccttgtaccagaagctcaagaaggtgggtcagggtgcgtctggctctgtgtatgtggctcggcctatgaccaacgagttccagctcaagaaggtggctatcaaacagatggatctggcgtcacagccccgaaaggagctcattgtgaacgaaatcatcgtcatgaaagagtcgcagcatgccaacattgtcaacttcctggacgcctatctgcgaggcaccaacgacctgtgggtggtcatggagtatatggagggcggtgctctcactgacattattgataacaacagtctgagtgaagagcagattgccacaatttccggcgagacctgtaaaggtctgcagcacttgcatcatcagaacattatccacagagacatcaagagtgacaacgtgctgttggactaccagggtcatgtgaagattactgatttcggtttctgcgccaagctcacggaccagaagaacaagcgagccaccatggtcggcacgccatactggatggcccctgaggtggtcaagcagaaggagtatggtgctaaggtagatgtgtggagcttgggtatcatggcgattgagatgatcgagtcagagcctccttacctgaacgaggagcctctcaaggctctgtacctgattgccactaacggtactcccaagctgaagcatcccgaacggctcagcaaggatattaaggcgttcctttctgtctgtctgtgtgttgatgtcaattacagagcctccaccgacgagctggtcaaccatgagttcttgaagaagggctgtggcttgcagagtttgagtccgctgttggcttacaagcataagaagatttag

**Reference**:

1. Holkenbrink C, Dam MI, Kildegaard KR, Beder J, Dahlin J, Domenech Belda D, et al. EasyCloneYALI: CRISPR/Cas9-Based Synthetic Toolbox for Engineering of the Yeast Yarrowia lipolytica. Biotechnol J. 2018;13(9):e1700543.
